# A social network perspective on peer relationship formation of medical undergraduates within large-scale learning communities

**DOI:** 10.1080/10872981.2022.2162253

**Published:** 2023-01-02

**Authors:** Yan Zhou, Nicolaas A. Bos, Agnes D. Diemers, Jasperina Brouwer

**Affiliations:** a Educational Technology, College of Teacher Education, Zhejiang Normal University, Zhejiang, China; b Center for Education Development and Research in Health Professions (CEDAR), LEARN, University Medical Center Groningen, University of Groningen, Groningen, The Netherlands; c Educational Sciences, Faculty Behavioural and Social Sciences, University of Groningen, Groningen, The Netherlands

**Keywords:** Learning community, informal peer network, collaboration, social network analysis, undergraduate medical education

## Abstract

**Introduction:**

Students’ formal networks, which are formed by a formal curriculum design, such as formally organized study groups within learning communities (LCs), may benefit students’ interactions and learning. It is unclear how large-scale LCs contribute to the formation of different informal peer relationships, which refers to student self-organized out-of-class relationships. Two mechanisms can explain relationship formation in LCs. Propinquity within formal networks and homophily of students’ characteristics (nationality, sex, academic performance) may promote students’ peer relationships. This study explores to what extent the formation of students’ informal networks was determined by their formal networks (LCs) while controlling for students’ characteristics and which mechanisms play an important role.

**Methods:**

With online surveys, data were collected about five informal networks (help-seeking, collaboration, information sharing, friendship, and learn-from) from 69 first- and 51 second- bachelor year medical students (2890 relationships). Students were divided into four LCs in the formal curriculum. We compared students’ five informal network structures between first- and second-year students, domestic and international students, within and between formal networks. Besides, we used Quadratic Assignment Procedure (QAP) Regression Analysis in Ucinet to investigate the associations between students’ informal and formal networks (LCs) and students’ characteristics.

**Results:**

Propinquity (in the same LC) plays a role since students have more informal connections within LCs than between LCs. Furthermore, it seems to play a greater role for second-year students than for first-year students. Homophily of nationality is important in informal networking since students are more likely to connect with others of similar nationalities.

**Conclusion:**

Students become more connected within the LC when they remain in the same LC for a longer period. Formal networks enhance the students’ informal interactions within LCs but seem to restrict the interactions among students from other LCs. International students need support in order to integrate with domestic students in LCs.

## Introduction

The development of society and the needs of patients are constantly challenging healthcare and medical education. Recently, many countries and universities focus on competency-driven medical education and consider local and global healthcare. As Frank et al. [1] mentioned, competency-based medical education (CBME) focuses on both clinical and educational outcomes, it allows for a highly personalized learning process instead of a traditional, one-size-fits-all curriculum [2], and provides a student-centered curriculum approach [1,3]. In CBME, students’ formal and informal peer networks are crucial to their learning. However, the diversity of formal network formations (size, duration) and student backgrounds leads to unclear results for various types of informal networks formation.

In the social learning theory Bandura et al. argued already more than half a century ago (1969) that students can learn within a social context, acquiring new information and behaviours by watching others [4]. Therefore, peer relationships are closely related to students’ learning processes and outcomes [5–9]. For the establishment of peer relationships both formal and informal networks seem to play an important role. Some universities consider using Learning Communities (LCs) to foster CBME implementation by creating students’ formal networks [10,11, 12–14] LCs are groups of students who share common academic goals and attitudes that meet regularly to collaborate on classwork, which benefits both their experience sharing, engagement, and professional competency development and interpersonal relationships [13,15,16]. The formal networks, which are usually organized by the faculty, offer a pre-structured environment where students do not have a choice with whom they collaborate. In addition to the formation of formal peer relationships, students will freely establish relationships with their peers out of class [17–20]. These are the so-called informal peer networks.

It is known that formal networks influence informal peer relationships formation to some extent. However, the diversity of formal networks formations and informal networks reveal conflicting results. On the one hand, formal networks benefit students establishing their informal relationships [6,19,21]. For instance, Champaloux et al. (2016) showed that small groups positively impacts peer selections for social interactions after class [20]. On the other hand, the small formal groups may not fulfill students’ social interactions, such as information sharing, so they prefer to form informal relationships with others out of formal networks [18,22–24]. Besides, formal networks may have different effects on different informal networks. Brouwer et al. (2018) found that students prefer to collaborate with others belonging to the same freshmen LC but this was not shown in friendship and help-seeking networks [24]. Thus, it is necessary to consider different types of informal networks (i.e., collaboration, information sharing) when investigating informal networks formation within the formal context of LCs.

As social interactions occur, several types of social relationships emerge. According to previous studies, five types of social relationships appear to be most relevant to the development of medical students’ competencies: (1) ‘study-related support’ (students seek help from others) [23,25–27]; (2) ‘collaboration’ (students collaboration with others for assignments or academic tasks) [24,28]; (3) ‘friendship’ [22,29,30]; (4) ‘information-sharing’ (students acquire or share information with others) [22,31]; and (5) ‘learn-from’ (students’ role model) [25,29]. Different peer relationships seem to be related, but research findings are ambiguous in this respect [23,24,28,29]. For instance, Stadtfeld et al. (2019) found that friendship and studying-together networks overlap and coevolve. This means that students are more willing to study or collaborate with their friends [23]. However, Zander et al. (2018) found that relationships between informal networks are not mutually associated [28]. Students who were initially more integrated with academic support networks became more integrated with social support networks over time, but not vice versa. Besides, it should be noticed that previous studies either included small (less than ten people) [18,20,21,25] to middle scaled (around 30 to 50 students) [18,28,30] formally structured groups when investigating informal networks. Few studies considered large scale formally structured organizations (i.e., LC more than 100 students) but not in medical education.,^34^

Besides the fact that group-size seems to affect peer network formation, also time may be an influencing factor on networks formation, although also in this respect the results seem to be ambiguous [22]. Some researchers found that the effect of the formally structured group on social and educational experience relationships intensified over time [19,20], whereas Hommes et al. (2014) found that the effect of formal network fades over time [22]. It is unclear how the effect of formal networks on informal networks varies between students with varying lengths of access to formal networks.

From social network research, it is well-known that two mechanisms can play a role in informal relationships’ formation [29,32]. The first mechanism is propinquity [33–35], which refers to the phenomenon that people are more likely to connect with others when they are physically close with each other [36]. The propinquity can be caused by formal networks, such as LCs, which may lead to more informal interactions within LCs rather than between LCs. Translating the findings of Borgatti and Cross (2003) to the educational context, students might seek information on the one hand, from an expert, but, on the other hand, go for ‘low costs’ and ask someone who is more available. Brouwer et al. (2018) showed indeed that social sciences’ students do not seek information from someone who is considered as an expert (a high-achieving student), but from a similar-achieving friend, which might be more convenient [8,28,37]. The second mechanism is homophily [35], which refers to the common finding that people are more likely to connect to each other based on similarities in their personal characteristics, such as academic performance, nationality [18,23,24,35,38]. For instance, in an ethnically diverse environment, students are more likely to connect with others according to similar nationality, ethnic background, or speaking language [39–41]. An homophily effect in terms of nationality may lead to boundaries between domestic students and international students and network segregation [30,39,41,42]. This is supported by previous studies which found that international students seem to have difficulties with connecting to domestic students both in and out of class [39–41]. Students may select each other based on the similarity of their background and this may create network segregation rather than the diversity for which educational designers often aim when they form their formal networks [24].

The existing body of knowledge about formal and informal networks is mostly derived from research in different higher education settings, but not so much specifically in medical education. With the increasing use of formal groups in medical education (e.g., small group, learning community) and the diversity of students’ backgrounds, we need to develop an in-depth understanding of the association between formal networks and informal peer networks formation. Additionally, since medical education programs are focusing more on collaborative learning but also more on self-study in recent years [43,44], it is relevant to consider different types of informal networks. In this study, we considered five informal networks (ask help, collaboration, share-information, friendship, and learn-from networks), which were demonstrated to relate to learning in previous studies [18,23,24,45], rather than two or three as been included usually [23,29]. Currently, we do not know how specifically medical students build different types of informal networks, the contribution of their characteristics (such as sex, nationality) and how large-scale formal networks influence these. Thus, in this study, we will investigate the impact of the formal network (LC) on the formation of five informal networks of medical students during the first two academic years. Considering the internationalization of medical education, we also compare informal connections between and within domestic and international medical students in our study. Based on the results, we provide suggestions to curriculum designers and administrators in medical schools to improve the learning process.

Thus, our research questions are:

1. What are the differences of structures between the five informal peer networks (study-related support, collaboration, share-information, friendship, and learn-from networks)?
2. To what extent was the formation of students’ informal networks determined by their formal networks (LCs)?
3. To what extent are background characteristics (sex, nationality, academic performance) and formal networks (LCs) linked to informal networks? To what extent are the five informal networks linked with each other?

## Methods

### Research setting

This study was conducted at a medical school in the Netherlands. To improve connections and collaborative learning, the medical school adopted four thematic learning communities (LCs): Sustainable Care (SC), Intramural Care (IC), Global Health (GH), and Molecular Medicine (MM). Each TLC has its own profile, content, task activities, learning materials, and assessments. The four LCs were considered as formal networks in this study. The LCs share a basic programme (same curriculum design and learning material) and have their LC specific task programme (different curriculum design and learning material). Each LC contains around 100 students. All students were full-time students and want to earn their degree at our medical school. LC SC and IC contain only domestic students and are taught in Dutch. LC GH and MM contain both domestic and international students and are taught in English. Students are assigned to one of the four LCs at the beginning of the first-year according to their interests and language preference. They stay in the same LCs for the first two and a half years of their three-year bachelor programme. Students within the same LC are allocated randomly into small tutor groups (of 10 students) every half-semester to facilitate propinquity (formal network influence). In tutor groups, students study and collaborate with others from the same LC. Tutor groups meet twice every week and are guided by trained faculty (tutors).

### Ethical approval

Ethical approval was obtained from the Ethical Review Board of the Netherlands Association of Medical Education (NVMO), dossier number 2019.4.9. The participation of students was voluntary and they consented to participate before answering the questions.

### Data collection and participants

First-year (*N* = 69, 16.4% of total first-year students) and second-year (*N* = 51, 13.0% of total second-year students) students participated in this study. For their characteristics see Table 1. The data were collected, at the end of the 2018/2019 academic year, through two approaches. First, we used an online questionnaire through Qualtrics to collect data about students’ informal networks. The questionnaire contained questions related to five informal networks: study-related support, collaboration, friendship, share-information, and learn-from (Table S1) [22,23,27]. The online questionnaire was distributed by email to all first- and second-year students in our medical school. After clicking the informed consent, we asked students to fill in fellow students’ names who they had regularly contacted in the past year out of class. Participants could list up to 35 fellow students. These fellow students’ names automatically appeared in the following questions related to informal networks so that students did not need to fill in their peers’ names again. They only need to click yes if they thought this student is the answer to the question.

**Table 1.** Demographics of the participants.

Characteristics of participants (N=120)		
	First year (BA1819)	Second year (BA1718)
LC	69	51
Sustainable Care (SC)	15	4
Intramural care (IC)	15	20
Global Health (GH)	19	12
Molecular Medicine (MM)	20	15
Female (%)	58 (84%)	39 (76%)
Domestic (%)	44 (64%)	42 (82%)
English-speaking LC (%)	39 (57%)	27 (53%)
Written test score average (SD)	6.323 (0.101)	6.061 (0.150)

We collected data about formal networks (LCs) and individual data (sex, nationality (domestic or international), written test scores) from the administration of the medical school (see also Table 1). In this study, we used the written test score as the grading system to present students’ academic performance, which is a curriculum-dependent test assessing medical knowledge. The four LCs used the same test questions so that we were able to compare students’ academic performance between four LCs. The range of test scores is from 0 to 10. The written test is performed by the students around five times every semester and we calculated the average score of all the taken tests.

### Data analysis

Social network analysis (SNA) techniques were used to investigate the impact of the formal peer network and individual characteristics on informal peer networks.

#### Data transformation

In this study, all informal networks and characteristics were transformed into dyadic variables. We used matrices to present the informal relationships and the relationship of characteristics between students. If students indicated the informal relationship, we assigned the value of ‘1’ in the matrix, otherwise the value of ‘0’ was assigned. Categorical variables (i.e., LC) or binary variables (i.e., sex, nationality (domestic or international), the language used in LC) were transformed by valuing ‘1’ if both individuals in the dyad were in the same category or valuing of ‘0’ otherwise. Continuous variables (i.e., written test scores) entered the models as absolute differences between individuals. In this case, the smaller the difference, the greater the similarity (homophily) in the dyad.

#### Whole network structures

To investigate the differences between the five informal peer networks for first- and second-year students in terms of network structures, we described and compared different informal networks’ constructions by calculating density, clustering coefficient, degree centrality, and reciprocity. The density refers to the proportion of the number of actual connections of possible connections in a network [46]. It indicates the association occurring between any pair of randomly selected students. The clustering coefficient presents the degree to which students in the network tend to cluster together [47]. It reflects the extent to which a network has areas of high and low density. If the clustering coefficient is much higher than the overall density, it indicates that some individuals are clustering in the entire network. Centrality is the construct we used to quantify the relatedness to other students in the social networks [48]. Degree centrality calculates the importance according to the number of links held by each student. It includes in-degree (number of inbound links) and out-degree (number of outbound links) [49]. The network level centrality presents the degree of inequality or difference in a network as a percentage of that in a perfect star network of the same size [50]. High centrality refers to the power of individuals with some individuals as the most influential or that they dominate the entire network. Reciprocity presents the likelihood of individuals reciprocating relations. It takes the number of reciprocated connections of students divided by the total number of connections [46].

#### The influence of students’ characteristics on their informal networks formation

To explore to what extent the formation of informal networks was determined by students’ characteristics, we calculate the E-I index concerning the nationality and sex. The E-I (external-internal) index measures the similarity of connected students by comparing the number of connections of students with the same and different characteristics [46]. It presents the similarity of students and their peers. In this study, we categorized students according to their characteristics (i.e., sex, nationality (domestic or international), formal group (LC)). To explore to what extent the formation of informal networks was determined by their formal networks (LCs), we calculated E-I index concerning the LCs and language used in LCs of five informal networks. The range of the E-I index is from −1 (all ties are internal to the group) to + 1 (all ties are external to the group) [50]. When the E-I index is less than 0, students tend to connect with others who have similar characteristics or are in the same formal groups.

#### Correlation among informal networks and influence factors

To investigate the associations between background characteristics (sex, nationality, academic performance), formal networks (LCs) and informal networks, and the associations among five informal networks, Quadratic Assignment Procedure (QAP) Correlation was used to analyze the statistical association between independent variables [46]. Independent variables include five informal networks (study-related support, collaboration, share-information, friendship, and learn-from networks) and their characteristics (i.e., sex, nationality (domestic or international), LC, written test score, language used in LCs).

All analyses were performed by using the UCINET 6.690 software package [46]. In addition, we used the Netdraw software package to visualize networks [46]. Figures describe the networks based on the characteristics.

## Results

RQ 1: What are the differences of structures between the five informal peer networks (study-related support, collaboration, share-information, friendship, and learn-from networks)?

### Whole network structures comparison

Considering the network structure, Table 2 shows the network density, cluster coefficient, centralization, and reciprocity of five informal networks. The density was almost similar in all networks of both first- and second-year students. This suggests that the degree of interconnectivity of all informal networks was almost similar. The clustering coefficient of informal networks differed between informal networks and also differed between first- and second-year students. We saw the highest clustering in the first-year students’ friendship networks. In general, first-year students showed more clustered networks than second-year students. Figure S1 and Figure S2 reveal this more intuitively.

**Table 2.** Descriptive statistics of network density, cluster coefficient, and centralization.

	Study-related support		Collaboration		Friendship		Share information		Learn from	
	First-year	Second-year	First-year	Second-year	First-year	Second-year	First-year	Second-year	First-year	Second-year
Network density	0.006	0.006	0.005	0.007	0.007	0.006	0.006	0.006	0.008	0.007
Clustering coefficient	0.090	0.060	0.053	0.067	0.107	0.052	0.071	0.039	0.057	0.027
Centralization										
Degree (Out)	7.68%	3.47%	4.90%	1.86%	10.68%	2.82%	10.64%	9.93%	14.36%	10.30%
Degree (In)	1.29%	0.42%	1.28%	0.26%	2.14%	0.41%	1.40%	1.43%	2.15%	1.27%
Reciprocity	0.064	0.040	0.044	0.052	0.086	0.040	0.068	0.031	0.056	0.019

Collaboration network showed much less centralization at the network level than the other four informal networks. High centrality refers to some individuals who are the most influential or dominate the entire network. Our result shows that collaboration is maybe more divided across all the students. This may be due to the random distribution of small groups in LCs. Students were able to collaborate with different fellow students in small groups over time. In general, all informal networks of second-year students were less centralized than first-year students. The reciprocity of friendship of first-year students was the highest. Literature shows that friendships are often based on mutual trustworthy relationships [26], it may explain the high reciprocity of friendship. The reciprocity of collaboration of second-year students was higher than first-year students. It may reflect the positive influence of peer relationships in the formal collaborative curriculum on informal collaboration networks.

### Informal networks formation concerning students’ nationality and sex

Table 3 shows the E-I index of informal networks concerning students’ characteristics. We found that the E-I indexes regarding nationality (domestic or international) of all informal networks are always lower than −0.50. When the E-I index is lower than 0, connections tend to be within the same nationality. The lower the E-I index, the more connection within the same nationality. It reveals that international students preferred to connect with other international students and domestic students preferred to connect with other domestic students. Second-year students had more study-related support and collaboration connections with others of different nationalities than first-year students, but less friendship, share information, and learn from connections with others of different nationalities. Besides, as Figure S1 and Figure S2 show, most connections across English taught and Dutch taught LCs were established by domestic students. Besides, students were more likely to connect with others of the same sex (E-I index <0, see Table 3). Except for the learn-from network, first-year and second-year students presented similarly on the E-I index for informal networks formation.

**Table 3.** E-I index of informal networks concerning students’ characteristics.

E-I Index	Study-related support		Collaboration		Friendship		Share information		Learn from	
Attributes	First-year	Second-year	First-year	Second-year	First-year	Second-year	First-year	Second-year	First-year	Second-year
LC	−0.511	−0.686	−0.775	−0.917	−0.500	−0.580	−0.549	−0.659	−0.607	−0.761
Sex	−0.557	−0.511	−0.486	−0.534	−0.485	−0.551	−0.527	−0.516	−0.374	−0.547
Nationality (domestic or international)	−0.720	−0.701	−0.695	−0.606	−0.596	−0.783	−0.600	−0.695	−0.579	−0.799
Language	−0.842	−0.839	−0.944	−0.938	−0.854	−0.761	−0.859	−0.812	−0.925	−0.874

RQ 2: To what extent was the formation of informal networks determined by their formal networks (LCs)?

### Informal networks formation concerning formal networks (four LCs)

To see whether students have more informal connections within LCs than between LCs, we calculated the E-I index concerning the LCs and language used in LCs of five informal networks (see Table 3). We found that the E-I indexes concerning LC of all informal networks were always lower than −0.50, which suggests that students had more informal connections with peers who came from the same LC than from different LCs. Besides, we noticed that E-I indexes concerning language are lower than E-I indexes concerning LC, close to −1, which means that although students had some connections across LCs, most of them occurred within two English LCs or within two Dutch LCs.

### Informal networks formation concerning formal networks (differences between first- and second-year students)

In addition, the E-I indexes concerning LC of second-year students are lower than those of first-year students, which suggests that second-year students generally had more connections within LCs than first-year students. Figure S1 and Figure S2 reveal this more intuitively. The friendship network, however, showed a similar E-I index of first-year and second-year students’ networks. In other words, second-year students seem not to establish more friendship ties within LCs than first-year students. In addition, second-year students showed slightly higher E-I indexes regarding language than first-year students. This means that even though the second-year students preferred to connect with fellow students within LCs, connections between English taught and Dutch taught LCs were slightly more than among the first-year students.

RQ 3: To what extent are background characteristics (sex, nationality (domestic or international), academic performance) and formal networks (LCs) linked to informal networks? To what extent are the five informal networks linked with each other?

### Correlation among informal networks and influence factors

To explore the association between five informal networks, and the association between informal networks and background characteristics and formal networks (LCs), we did a QAP correlation analysis [46]. We found moderate to strong positive correlations between the five networks (study-related support, collaboration, share-information, friendship, and learn-from networks) for both first- and second-year students. The collaboration networks of second-year students were more strongly correlated with other networks than those of first-year students. It reflects that second-year students’ other informal peer networks (study-related support, friendship, share-information, and learn-from networks) are more influenced by collaboration networks than first-year students. In contrast, background characteristics and formal networks showed very weak correlations with the five informal networks. Furthermore, the correlation between background characteristics and informal networks was stronger in the cohort of first-year students than in the cohort of second-year students (see Table 4).

**Table 4.** QAP correlations between networks and students’ characteristics.

	Variables	1	2	3	4	5	6	7	8	9	10
**First-year**											
1	Study-related support	1.000									
2	Collaboration	0.614*	1.000								
3	Friendship	0.766*	0.543*	1.000							
4	Share information	0.720*	0.594*	0.762*	1.000						
5	Learn from	0.578*	0.456*	0.621*	0.617*	1.000					
6	LCs	0.078*	0.079*	0.080*	0.079*	0.063*	1.000				
7	Nationality (domestic or international)	0.035*	0.027*	0.026*	0.026*	0.018*	0.141*	1.000			
8	Sex	0.019*	0.011*	0.014*	0.016*	0.003	−0.002	0.064*	1.000		
9	Written test	−0.021*	−0.016*	−0.018*	−0.020*	−0.015*	−0.019*	0.031	−0.047	1.000	
10	Language used in LCs	0.056*	0.050*	0.058*	0.055*	0.045*	0.580*	0.246*	0.006	−0.016*	1.000
**Second-year**											
1	Study-related support	1.000									
2	Collaboration	0.689*	1.000								
3	Friendship	0.737*	0.604*	1.000							
4	Share information	0.690*	0.642*	0.770*	1.000						
5	Learn from	0.651*	0.572*	0.602*	0.647*	1.000					
6	LCs	0.006	0.010*	0.004	0.002	0.010*	1.000				
7	Nationality (domestic or international)	−0.008	−0.003	−0.004	−0.004	0.005	0.202*	1.000			
8	Sex	−0.009	−0.003	−0.009	−0.008	−0.002	0.003	0.022	1.000		
9	Written test	0.007	0.013*	−0.013*	0.012	0.010	0.002	−0.003	−0.024	1.000	
10	Language used in LCs	0.015*	0.015*	0.011*	0.009*	0.017*	0.588*	0.316*	0.011	0.003	1.000

Significance levels: *p ≤ 0.05. All variables are at the dyadic level.

## Discussion

This study uses the social network analysis method to present the effect of the formal peer network (LC) on informal peer network formation (study-related support, collaboration, share-information, friendship, and learn-from networks) considering student characteristics (i.e., nationality, sex and academic performance). We first investigated the differences between the five informal peer networks for first- and second-year, domestic and international medical students in terms of network structures. Then we inquired to what extent the formation of students’ informal networks was determined by their formal networks (LCs) and students’ background characteristics (sex, nationality, academic performance), and to what extent the five informal networks are linked with each other.

The result of QAP correlation analysis supports the literature showing the association between the five informal networks(study-related support, collaboration, share-information, friendship, and learn-from networks), and the relationship between characteristics(i.e., nationality, sex, academic performance, formal networks (LC) and informal networks [22,23,39–41]. Propinquity (stay in the same LC) plays a role since students have more informal connections within LCs than between LCs, which is consistent with previous findings [18,20]. Formal networks (LC) enhance the possibility of informal relationship formation since students may easily know each other and start to form informal relationships [20]. Besides, we found that the five informal peer networks, such as friendship and share information, are positively correlated with each other. It may mean that when students collaborate, they are also likely to become friends, seeking academic help, sharing information and learn from each other [23,51]. Background characteristics (sex, nationality, academic performance) are only weakly related to informal networks, and the similarity of characteristics is hardly associated with the formation of informal networks in contrast to other studies who found that homophily can be important in informal networking [35,52].

Furthermore, we explored the differences of informal networks considering the influence of formal networks and the length of students were in the same LC. It contributes to clarifying the role of lengths of access to formal networks and personal characteristics in informal peer relationships. Our finding that the social and educational experience relationships are more clustered when they stay longer in the college system is consistent with earlier research [19,20,29] but contradicts the findings of Hommes et al. (2014) who found that the formal network’s influence on friendship and information sharing network fades over time [22]. In the longitudinal study of Hommes, the formal networks were tutorial groups, which included 10 to 12 students in each group that were followed over time [22]. It was different from this study which is cross sectional and involves larger formal networks such as LCs, thus, more research is needed about the effects of different group sizes and time on peer relationship formation. Besides, personal characteristics are more important for first-year students than second-year students in informal network formation because they meet each other for the first time and will contact others according to visible attributes [53]. It reflects the importance of formal networks on their informal networks on a long-term basis. Therefore, if we want to promote informal interactions among students with different characteristics, we can place students in a formal network for a longer time. Although students preferred to connect with others who are similar to them in informal networks, the similarity of characteristics cannot predict the relationship vice versa.

Different from other informal networks, however, when comparing structure of networks, friendship networks did not show much-increased internality (more connections within LC) in second year students compared to first year students. In contrast, students have more collaboration relationships within LCs than other informal peer relationships. The differences in the curriculum design of the four LCs may be the reason. Students may prefer to collaborate with others who face similar problems or tasks especially. Therefore, the differences between LCs may be also the explanation for the dissimilarity of friendship and collaboration networks. It slightly differs from Zander’s finding that students who are more integrated with academic support networks became more integrated with social support networks over time [28]. Considering the positive influence of formal networks on informal collaboration and the positive association between informal peer relationships, it is worthwhile for medical schools to provide more opportunities to increase the propinquity (collaborate in small groups in the formal curriculum), which may foster the training of excellent team collaborators [54].

Moreover, we explored the differences in informal networks formation between international and domestic students. The connections of informal networks between domestic and international students are much less than within domestic students or within international students, specifically across LCs, which is consistent with previous studies [30,39,42]. The English taught LCs mixing domestic and international students did improve informal peer relationships between domestic and international students. However, most students’ connections between English and Dutch taught LCs were established by domestic students. This may be explained by language. Since international students are inadequately able to speak Dutch, especially in the first year, LCs using English may benefit international students’ communications with domestic students. Extra support for international students is necessary if we want to enhance their connections with domestic students, specifically to domestic students from Dutch LCs.

## Strengths, limitations, practical implications and future research

The first strength of this study is that we investigated five types of informal networks. These five peer relationships are different itself. Previous research did a lot in comparing collaboration, friendship, and help-seeking, we took learn-from and share-information into account as well in this study [23,24,29]. We were able to explore the relationship between different types of informal networks, which adds to the already existing knowledge. Second, this is one of the first studies that address the effect of large-scale formal networks in terms of LCs on undergraduate informal peer networks formation within the medical field. It contributes to the diversity of literature regarding the effect of formal networks on informal peer relationships and to the understanding of the effect of formally structured organizations on peer relationships. Third, considering the globalization of medical education, we investigated the differences between domestic and international students’ informal networks formation. Our results may help curriculum designers to develop their programme design in such way that international students have more opportunities to be involved in different kinds of informal networks.

We consider three important limitations of our study. First, this study is a cross-sectional study, which means that we only captured informal networks at one-time point. We were unable to follow the development of the informal network over time and we cannot make any inferences about causality [55]. Future research could consider a longitudinal social networks study to make it clear how informal peer relationships develop over time [51]. Second, the response rate of this study is not that high. Students who have less contact with others might be less inclined to take part in such investigations. However, the distribution of students from different LCs is relatively balanced. Third, we took a quantitative approach to address the question to what extent students are connected. We do not address the question of why students connect. Therefore, future research could consider qualitative research (i.e., interview) or mixed-method research to investigate how students build their informal networks, and try to involve more participants, gaining insight into the role of formal networks in forming informal peer relationships within medical curricula. Fourth, we only consider the association between informal networks and three objective characteristics of students (sex, nationality, academic performance) in this study, but more objective characteristics, for instance economic and social status, entry performance, and subjective characteristics, such as motivation, self-efficacy, emotions (anxiety, belonging) may also influence their informal relationships [56–58]. Future studies may shed a light on how other objective and subjective characteristics influence informal peer network formation.

## Conclusion

This study addresses the effect of formal networks on students’ informal networks formation within the medical field. Propinquity plays a role since students have more informal (collaborative) connections within LCs than between LCs. Formal networks (study groups organized by faculty) and various curriculum design in four LCs enhance the informal interactions within LCs but seem to restrict the interactions among students from other LCs. The LC seems to play a greater role in the informal peer network formation for second-year students than for first-year students. Homophily is important in informal networking since students are more likely to connect with others of similar nationalities. International students need more support to connect with domestic students, especially between LCs, due to fewer ties between nationalities than within nationality.

## Supplementary Material

Supplemental Material

## Data Availability

The dataset used and analyzed during this study is available from the corresponding author upon reasonable request.
