# Supplementary material for: A social network perspective on peer relationship formation of medical undergraduates within large-scale learning communities
Source: Med Educ Online. 2023 Jan 2;28(1):2162253. doi: 10.1080/10872981.2022.2162253 (PMC9815217; doi:10.1080/10872981.2022.2162253)
Supplement: Supplemental Material [file ZMEO_A_2162253_SM9449.zip › Supplementary files/Supplementary Figures.docx]

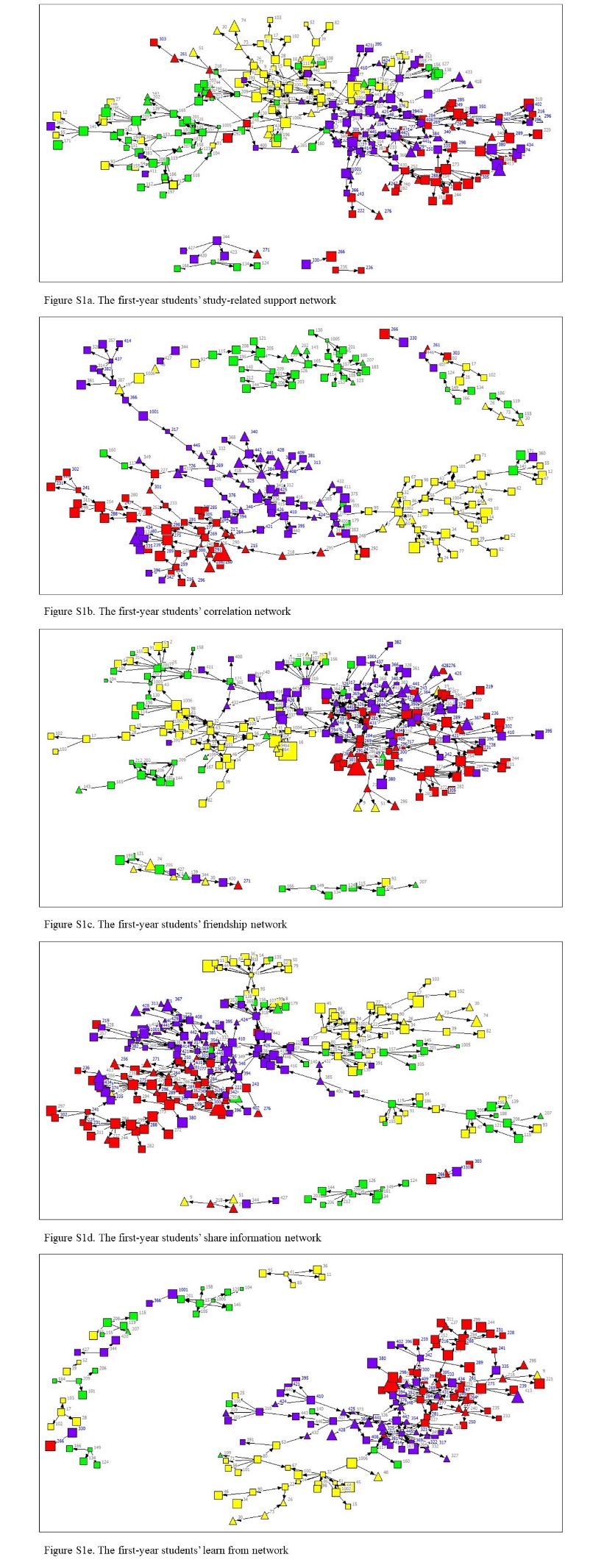


**Figure S1.** First year students’ networks. Each node presents a student. The color of the nodes presents student’s learning community (yellow = LC SC, green = LC IC, red = LC GH, purple = LC MM). LC SC and LC IC are taught in Dutch, and LC GH and LC MM are taught in English. The shape of the nodes presents student’s sex (square = female, up triangle = male). The size of the node presents the written test score. The bigger size, the higher score. The color of the label presents students’ countries of origin (blue = international students, gray = domestic students).


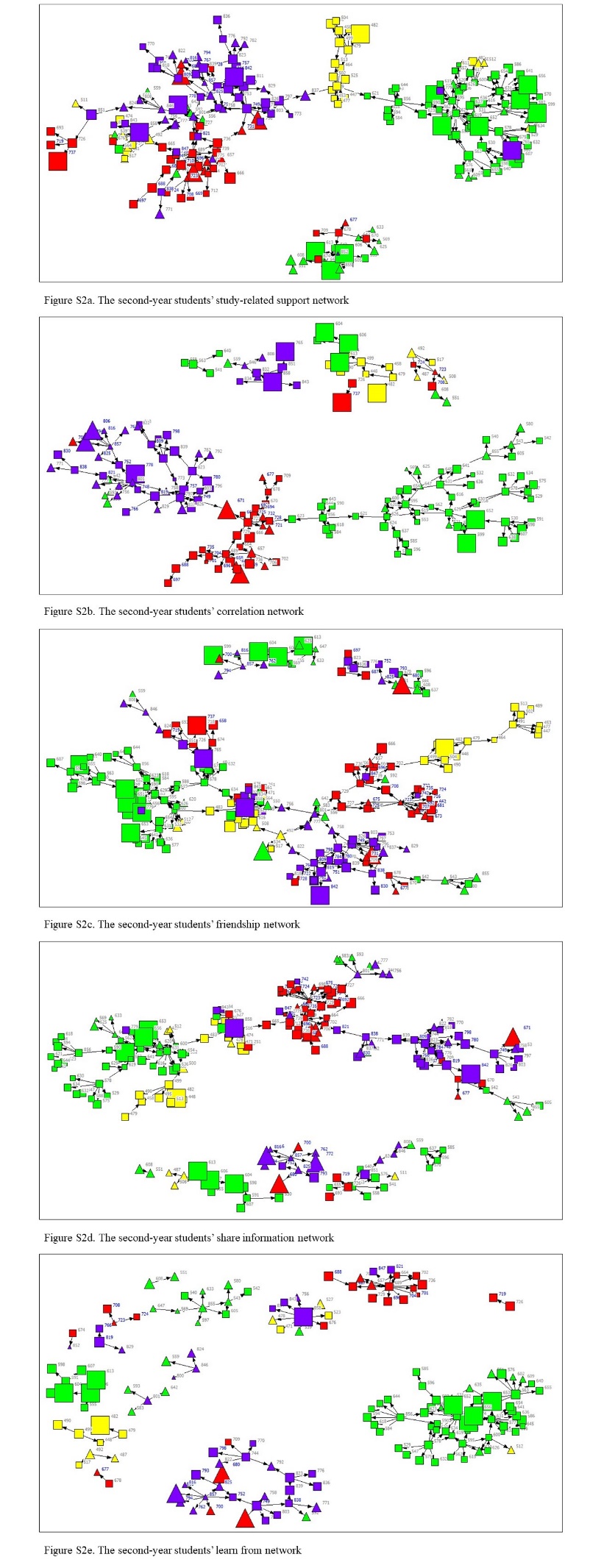


**Figure S2.** Second year students’ networks. Each node presents a student. The color of the nodes presents student’s learning community (yellow = LC SC, green = LC IC, red = LC GH, purple = LC MM). LC SC and LC IC are taught in Dutch, and LC GH and LC MM are taught in English. The shape of the nodes presents student’s sex (square = female, up triangle = male). The size of the node presents the written test score. The bigger size, the higher score. The color of the label presents students’ countries of origin (blue = international students, gray = domestic students).
