# Supplementary material for: A social network perspective on peer relationship formation of medical undergraduates within large-scale learning communities
Source: Med Educ Online. 2023 Jan 2;28(1):2162253. doi: 10.1080/10872981.2022.2162253 (PMC9815217; doi:10.1080/10872981.2022.2162253)
Supplement: Supplemental Material [file ZMEO_A_2162253_SM9449.zip › Supplementary files/Table S1.docx]

**Table S1.** Questions about five informal networks in the online questionnaire

| network | Question |
| --- | --- |
| Study-related support | “In the past year, when I didn’t understand the study material, I shared my questions or problems that I faced, with this fellow student [name] outside of class.’’ |
| Collaboration | “In the past year, I collaborated with the following fellow students [name] to complete assignments/tasks outside of class.’’ |
| Friendship | “whom [name] would you classify as your friend from your fellow students.’’ |
| share Information | “In the past year, I shared study-related information/data with these fellow students [name] outside of class.’’ |
| Learned from | “During the past year, I have learned a lot from [name].’’ |
